# Signs and symptoms of foot and ankle dysfunction in children with joint hypermobility

**DOI:** 10.1186/1757-1146-7-S1-A61

**Published:** 2014-04-08

**Authors:** Leslie L Nicholson, Verity Pacey, Louise Tofts, Craig Munns, Roger Adams

**Affiliations:** 1 Discipline of Biomedical Science, Sydney Medical School, The University of Sydney, Australia; 2 Physiotherapy Department, The Children’s Hospital at Westmead, Sydney, Australia; 3 Kids Rehab, The Children’s Hospital at Westmead, Sydney, Australia; 4 Discipline of Paediatrics and Child Health, Sydney Medical School, The University of Sydney, Sydney, Australia; 5 Endocrinology Department, The Children’s Hospital at Westmead, Sydney, Australia; 6 Discipline of Physiotherapy, Faculty of Health Sciences, The University of Sydney, Sydney, Australia

## Background

Foot and ankle complaints are common in people with hypermobility. Children with hypermobility (Beighton score ≥4/9) were recruited for a longitudinal study from The Children’s Hospital at Westmead in Sydney, Australia. Baseline data included Beighton score, BMI for age, Star Excursion Balance Test (SEBT), Foot Posture Index (FPI), anterior drawer, subtalar inversion stress test, Lower Limb Assessment Scale (LLAS), physical activity and child-rated quality of life (PedsQL).

## Results

53 girls and 47 boys (mean age 11.5±3.1yrs) with a mean Beighton score of 6.7/9 and LLAS of 8.2/12 were recruited. Of these 100 children, 94 met the Brighton criteria for Joint Hypermobility Syndrome [1] and 90 met the Villefranche criteria for Ehlers-Danlos Syndrome-Hypermobility Type [2]. Of the entire cohort, 50% reported experiencing ankle joint pain and 13% foot pain that had lasted 3 or more months, 36% reported recurrent “rolling” one or both ankles while only 8% reported foot instability. The average FPI in this cohort was +6.6, with 86% of the children having FPI scores of 5 or more and 19% with scores of 10 or more. Paired samples t-tests revealed that those children who reported chronic ankle pain were the ones experiencing recurrent episodes of instability (p=0.016). Recurrent instability did not significantly correlate with anterior talofibular ligament laxity as assessed with the ankle anterior drawer test or the subtalar inversion test or with foot posture (all p>0.5). While the Beighton score moderately correlated [3] (r=0.31, p= 0.002) with the LLAS, only the LLAS correlated with physical activity (r=-0.29, p=0.005). The SEBT and BMI for age correlated moderately (r=0.4, p<0.001; r=-0.31, p=0.003) with child-rated quality of life.

## Conclusion

Half of the hypermobile cohort in this study reported experiencing chronic ankle pain which was associated with recurrent episodes of instability. Interestingly, instability and laxity were not correlated in these children suggesting that instability may be neuromuscular in origin. The LLAS may provide more valid quantification of the extent that lower limb joint hypermobility affects physical activity than the more commonly used Beighton score in these children.
